# Can Early Nutrition Be Responsible for Future Gut Microbiota Changes and Different Health Outcomes?

**DOI:** 10.3390/nu17233721

**Published:** 2025-11-27

**Authors:** Raffaella de Franchis, Luigi Bozza, Paolo Cortese, Lorenzo D’Antonio, Antonio D’Avino, Nicoletta Gasparini, Giorgia Ippolito, Raffaella Spadaro, Mariangela Tedesco, Angelo Antignani, Francesca De Filippis, Vincenzo Valentino, Renata Auricchio, Salvatore Auricchio, Dario Bruzzese

**Affiliations:** 1 Italian Federation of Maedical Paediatrics (FIMP), 80142 Naples, Italy; luigibozza1963@virgilio.it (L.B.); pacortese@libero.it (P.C.); davinoant@gmail.com (A.D.); nicolegasparini@libero.it (N.G.); raffyspadaro@gmail.com (R.S.); tedescomariangela@alice.it (M.T.); 2 Department of Translational Medical Sciences, University of Naples Federico II, 80131 Naples, Italy; lorenzodantonio5@gmail.com (L.D.); r.auricchio@unina.it (R.A.); 3 Department of Woman, Child and of General and Specialized Surgery, AOU University of Campania Luigi Vanvitelli, 80138 Naples, Italy; gio.ippolito94@gmail.com (G.I.); dr.angeloantignani@gmail.com (A.A.); 4 Department of Agricultural Science, University of Naples Federico II, 80131 Naples, Italy; francesca.defilippis@unina.it (F.D.F.); vincenzo.valentino2@unina.it (V.V.); 5 European Laboratory for the Investigation of Food-Induced Diseases, University of Naples Federico II, 80131 Naples, Italy; salauric@unina.it; 6 Department of Public Health, University of Naples Federico II, 80131 Naples, Italy; dario.bruzzese@unina.it

**Keywords:** Mediterranean diet, complementary feeding, chronic inflammatory diseases, gut microbiota, public health

## Abstract

**Background/Objectives**: Chronic inflammatory diseases (CIDs) often present a preclinical phase influenced by genetic and environmental factors, including nutrition. Early dietary habits may modulate long-term health trajectories by shaping the intestinal microbiota. Previous work showed that weaning with fresh foods from the Mediterranean diet (MD) improved dietary habits and microbiota composition at 3 years of age. This study aimed to assess whether such benefits persist at 9 years. **Methods**: This long-term follow-up included 191 children (96 MD, 95 controls) from the original randomized cohort (ClinicalTrials.gov NCT05297357). The primary endpoint was adherence to MD (KidMed score ≥ 8). Secondary endpoints included BMI, incidence of CID, maternal dietary adherence, and intestinal microbiota composition in a subset of 36 children. **Results**: At 9 years, no difference was found in overall MD adherence (27.4% controls vs. 27.1% MD; *p* > 0.99) or BMI (17.7 vs. 18.1 kg/m^2^; *p* = 0.384). However, children from the MD group reported higher daily vegetable intake (71.9% vs. 51.6%; *p* = 0.005). Microbiota analyses revealed persistent differences between groups, with higher alpha diversity in the MD group. Although not statistically significant, the MD group showed lower prevalence of atopic dermatitis, allergic rhinitis, autism spectrum disorder, and ADHD. Maternal adherence to MD did not differ between groups. **Conclusions**: Early introduction of Mediterranean-style foods during weaning exerts lasting effects on dietary patterns and gut microbiota, with a potential protective trend against CID. While overall MD adherence converged between groups by 9 years, these findings suggest that early-life nutritional interventions may induce durable microbiome-mediated benefits and contribute to preventive strategies for chronic disease, warranting confirmation in larger, extended cohorts. Moreover, this study highlights the value of the collaboration between the Italian primary care pediatric system and the integration of the pediatric residency program, demonstrating a feasible and cost-effective methodology to generate large-scale prospective data within routine clinical practice. Larger studies and a longer follow-up period are necessary to confirm these results.

## 1. Introduction

Chronic inflammatory diseases (CIDs) are increasing throughout the Western world, affecting individuals across all age groups [1]. Recent studies have shown that some CIDs may have a preclinical phase that can be detected through specific biochemical markers [2]. These findings support the hypothesis that the true onset of certain diseases occurs many years before the manifestation of clinical symptoms, suggesting that preventive action should ideally begin in the earliest stages of life. Crohn’s disease and ulcerative colitis are currently the most extensively studied conditions in this regard, but evidence of a preclinical phase has also been reported for type 1 diabetes and rheumatoid arthritis [3]. The onset of these diseases is thought to result from the interaction between multifactorial genetic susceptibility and environmental factors, including infections and nutrition.

At present, no primary or secondary prevention strategies or official recommendations exist for healthy individuals at high risk of developing inflammatory bowel disease (IBD), such as those with a family history. Nevertheless, potential preventive measures have been suggested, including promoting breastfeeding, smoking cessation, minimizing antibiotic exposure, regular physical activity, and adopting dietary patterns that avoid ultra-processed foods while encouraging adherence to a Mediterranean-style diet [3].

The benefits of the Mediterranean diet (MD)—such as reductions in plasma lipids, inflammatory markers, and oxidative stress; improvements in insulin sensitivity and endothelial antithrombotic function; and a slowing of neuronal degeneration—have been highlighted in numerous studies [4,5]. In contrast, a Western-style diet, characterized by high intake of dairy products, food additives, simple sugars, animal fats, and salt, and low consumption of dietary fiber of plant origin, has been associated with an increased glycemic load and elevated intake of saturated fatty acids. These factors have inevitable negative effects on human health, partly mediated through alterations in the intestinal microbiota and the promotion of systemic low-grade inflammation [6]. In a previous study [7], we demonstrated that early nutritional intervention—specifically, weaning at 4–6 months of age using fresh, adult foods typical of the Mediterranean diet—was able to modify the eating habits of the treated children and their families. Moreover, beneficial changes in the gut microbiota were observed in a subgroup of treated children. These findings highlighted the importance of investigating the long-term impact of interventions that are both effective and easily implemented in pediatric practice, such as dietary guidance at weaning. They also provided a model for studying the longitudinal effects of early interventions (e.g., diet at weaning).

Here, we present a long-term follow-up of the original cohort to assess the effects, after 9 years, of different weaning strategies on children’s and families’ dietary habits, intestinal microbiota, obesity, and the development of CID.

## 2. Materials and Methods

This study is a long-term follow-up of the trial on Med Diet registered at ClinicalTrials.gov (NCT05297357). The original trial verified the effects of weaning using adult foods typical of the Med Diet on eating habits and the gut microbiota composition of children [7]. No special educational counseling on nutrition was carried out by the family pediatricians who took care of the children from the age of 3 years up to the evaluation time, either in the MD group or in the control group. Adherence to the Med Diet by all children and their mothers was investigated as already described [7]. The presence of a chronic inflammatory disease, as well as the BMI, was evaluated in all children to verify the development of obesity.

We also analyzed the intestinal microbiota in a subset of 36 patients (15 cases and 21 controls) of the original 51 patients, already tested at the beginning of our study [7].

### 2.1. Aims

The primary objective of this follow-up study was to evaluate the impact of a weaning approach based on fresh, seasonal, and natural foods on children’s long-term adherence to the Mediterranean diet (MD).

Secondary objectives included assessing the effects of this weaning model on children’s BMI at nine years of age, the cumulative incidence of non-communicable diseases during follow-up, mothers’ adherence to the MD, and the gut microbiota composition of the children.

### 2.2. End Points

Primary and secondary endpoint assessment refers to the fifth follow-up occasion, which was planned once children reached 9 years of age. The primary efficacy endpoint was the percentage of children showing good adherence to the MD, defined as a score in the KidMed questionnaire ≥8.

Secondary endpoints were the average children’s BMI, the average mother’s adherence to MD, the cumulative incidence of non-communicable chronic diseases, and gut microbiota composition in kids weaned using the MD and kids weaned using traditional schemas.

Definition of overweight and obese children and the evaluation of adherence to MD by children and their mothers were performed as already described [7].

### 2.3. Statistical Analysis

All statistical analyses were conducted using the platform R (vers. 4.3; The R Foundation for Statistical Computing, Vienna, Austria).

Demographic and clinical data were summarized using standard descriptive statistics; between-group differences were assessed either using *t*-test for independent samples or the Fisher exact test. Correlation between the BMI and KidMed scores was evaluated using the Pearson Correlation Coefficient.

Differences in the overall microbiota composition between the two groups were evaluated by Permutational multivariate analysis of variance (nonparametric MANOVA) based on the Bray–Curtis distance matrix. Pairwise Wilcoxon tests were used to determine significant differences in alpha diversity parameters or in the abundance of specific taxa at each sampling occasion (at three and nine years of age).

In all analyses, a *p*-value < 0.05 was considered statistically significant. No adjustment for multiple comparisons was applied.

### 2.4. Fecal Microbiota Analysis

Fecal sample collection and total microbial DNA extraction were performed according to the Standard Operating Procedure 04 (SOP04) and SOP07 (version 2) by the International Human Microbiome Standard (IHMS) Consortium, respectively. For each sample, the V3–V4 hypervariable region of the rRNA 16S gene was amplified through a Polymerase Chain Reaction (PCR), using primers S-D-Bact-0341-b-S-17 and S-D-Bact-0785-a-A-21, as previously described [8]. Sequencing was carried out on Illumina MiSeq platform, leading to 2 × 250 bp reads. Sequences were processed with QIIME2 q2cli v2020.04, and the plugin DADA2 was used to trim primers and low-quality bases, as well as denoise and merge forward and reverse reads. Chimeric sequences were found and filtered through the options “—p-chimera-method pooled” and “—p-min-fold-parent-over-abundance 10”.

Taxonomy was inferred by mapping the representative sequences against the Greengenes database (release 13_8) through the ‘consensus_vsearch’ method included in the ‘feature-classifier’ plugin.

The resulting ASV (Amplicon Sequence Variants) table was imported into an R environment (version 4.3) for statistical analysis and visualization. Samples were rarefied at the same number of sequences; then, alpha-diversity indices were estimated.

## 3. Results

Of the original sample of 394 children enrolled from May 2015 to July 2016 [7], data on 191 children were available for this post hoc analysis (95 controls and 96 originally in the MD arm, Table 1) who were followed longitudinally up to June 2024.

### 3.1. Mediterranean Diet Adherence

No difference in the percentage of children showing good adherence to the MD was noted between the two groups of at 9 years of age: 27.4% vs. 27.1 in the control and MD group, respectively (95% CI for the between group difference: −13.2%; +12.6%, *p* > 0.999). The average Kidmed score was also similar in the two groups: 5.7 ± 2.3 vs. 6.1 ± 2.3 in the control and MD group, respectively (*p* = 0.274).

However, with respect to the single items of the KidMed questionnaire, children in the MD group showed higher levels of regular consumption of fresh and cooked vegetables per day (51.6% vs. 71.9%; *p* = 0.005) (Table 2).

Mothers’ adherence to the Med Diet was evaluated when children reached 9 years of age. No statistical difference was noted in the mothers of the two groups of children (68% ± 14% vs. 71.1% ± 14.3%; *p*=0.199).

### 3.2. Anthropometric Measurements

**Figure 1:**
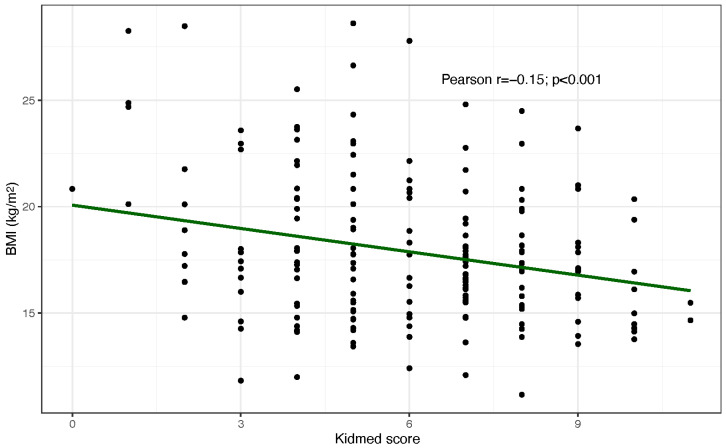
Correlation between BMI and Kidmed score in the overall sample.

No significant differences between the groups were observed with respect to BMI: 17.7 ± 3.5 kg/m^2^ in controls and 18.1 ± 3.4 kg/m^2^ in the MD group; *p* = 0.384. The percentage of obese children was lower in the MD group although this difference did not reach statistical significance (Table 3), and a weak but significant negative correlation between KidMed adherence and BMI was observed (Figure 1).

### 3.3. Non-Communicable Inflammatory Chronic Diseases

At 9 years of age, children were assessed for Non-Communicable Chronic Inflammatory Diseases. Although no significant difference emerged between the two groups, a higher prevalence of some conditions was noted in the control group. Of the 27 children presenting either Atopic Dermatitis (AD) or allergic rhinitis, only 11 were present in the MD group (13.4% vs. 19.3%; *p* = 0.401). A higher prevalence of autism spectrum disorders (4.8% vs. 0%; *p* = 0.12) and ADHD (4.8% vs. 2.4%; *p* = 0.682) was also observed in the control group. When pooling the two diseases, the prevalence was 9.6% vs. 2.4%, respectively (*p* = 0.099) (Table 4).

### 3.4. Gut Microbiota Composition

Gut microbiota composition was evaluated in 36 children (15 MD and 21 control) from the original cohort [7] at approximately 9 years of age.

**Figure 2:**
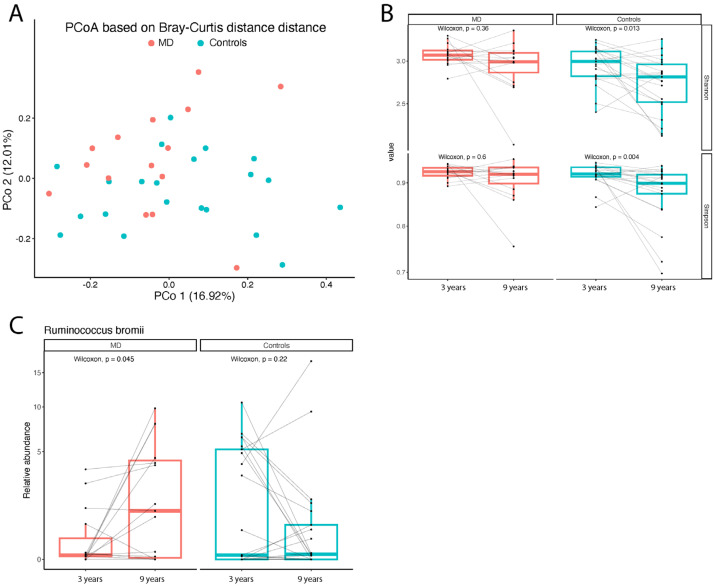
Analysis of gut microbiota composition. (**A**) PCoA plane based on Bray–Curtis distance showing differences in the intestinal microbiota between MD and controls at nine years. Each point represents a sample. (**B**) Boxplots showing the longitudinal within-group alpha diversity of the gut microbiota. Each point represents a sample, and the horizontal gray lines connect samples from the same subject. (**C**) Boxplot comparing the relative abundance (%, on the *y*-axis) of *Ruminococcus bromii* between samples collected from the same subjects at different timepoints (3 and 9 years). Each point represents a sample. The horizontal gray lines connect samples from the same subject.

The intestinal microbiota (IM) profiles of the intervention group were compared with those of the controls, and both were also evaluated against the corresponding profiles of the original 51 children from which this subsample was drawn. As in the original study, a significantly different gut microbiota composition between MD and control groups was confirmed (PERMANOVA, *p* = 0.015; Figure 2A). Comparison of biodiversity indices in the subsample of 36 children, with the original sampling at 3 years of age, showed that biodiversity remained stable in the MD group, whereas it decreased in controls (Figure 2B). Longitudinal analysis within the MD group revealed an increase in the short-chain fatty acid (SCFA) producer *Ruminococcus bromii* (Figure 2C), along with a significant rise in several taxa of the Lachnospiraceae family, also known as SCFA producers. 

Of note, when these results were compared with those previously reported [7], the set of significantly different species was not the same. Although the most abundant taxa identified at 3 years were no longer predominant at 9 years, a distinct representation of beneficial species persisted in the intervention group.

## 4. Discussion

In a previous study [7], we demonstrated that an early dietary intervention—introducing fresh, home-cooked Mediterranean diet (MD) foods at weaning (4–6 months) in a palatable manner—successfully influenced the eating habits of both treated children and their families at 3 years of age. Beneficial modifications in the gut microbiota were also observed in the intervention group.

The present follow-up study assessed the long-term impact of this early intervention on children’s and families’ dietary habits, as well as on children’s gut microbiota composition, up to the age of 9 years. A secondary objective was to evaluate whether the intervention reduced the incidence of CID.

At 9 years of age, no significant differences in overall adherence to the MD were observed between the intervention and control groups, nor among their mothers, in contrast to our earlier findings at 3 years [7]. However, children in the intervention group continued to consume fresh, cooked vegetables daily. Despite similar dietary patterns at 9 years, the gut microbiota composition differed between groups: the intervention group retained a higher prevalence of beneficial microbial species, although the specific bacterial taxa differed from those observed at 3 years. Other authors demonstrated that a critical window exists during early-life microbial development, considering early nutrition one of the main drivers influencing microbiota reprogramming [9]. An eubiotic gut ecosystem is protective against several diseases by facilitating correct gut axis connection to key distant organs like brain, lungs, skin, adipose tissue, and pancreas. From this perspective, our findings support the hypothesis that gut microbiome development through the three crucial stages up to 46 months (early, transitional, and stable phases) [10] is influenced by early-life exposure to a healthy diet, which induces a lasting “priority effect” on the microbiome that remains beneficial over time. This mechanism has already been hypothesized by some authors [5], even if experimental studies in the first years of life are very few. Our experimental group of children was weaned using fresh foods typical of the MD and prepared in a palatable way. It is possible that early exposure to such foods facilitated the development of an eubiotic gut eco system in this group of children. This hypothesis is supported by the longitudinal increase in *R. bromii* in the intervention group, as this species can degrade resistant starch [11], a type of fiber commonly found in whole grains typical of the MD [12].

In addition, the higher daily consumption of fresh and cooked vegetables observed in our intervention group at 9 years of age may partly explain the maintenance of a eubiotic state over time—that is, the persistence of beneficial taxa despite a diet no longer strictly adhering to the MD at 9 years of age. Indeed, we observed stable gut microbial diversity in the intervention group, as opposed to the drop in biodiversity observed in controls at 9 years of age.

The interplay between diet, gut microbiota, and health outcomes has been extensively reviewed by Ross et al. [13], who highlighted how dietary patterns influence the stability, functionality, and diversity of the intestinal microbiome, and how nutritional modulation could help prevent or delay the onset of chronic diseases. In this context, our findings provide evidence that dietary interventions during the first years of life may reduce baseline inflammation and the risk of intestinal damage. Similarly, breastfeeding has been shown to increase early microbiome diversity, bringing it closer to that of adults [13].

Regarding CID incidence at 9 years, no statistically significant differences were detected between groups. Nevertheless, the control group showed a higher prevalence of atopic dermatitis, allergic rhinitis, autism spectrum disorder, and ADHD compared with the MD group, suggesting a potential protective trend associated with early introduction of the MD, possibly due to the early development of a proper relationship between an eubiotic gut system and the axes connected to distant target organs (brain, lungs, skin, adipose tissue, and pancreas). In contrast, children who were not exposed to healthy foods of the MD in the early stages of life show a higher incidence of CID due to early gut dysbiosis. As for obesity, no differences were observed between groups, in line with our previous results [7]. However, higher adherence to the MD was associated with lower BMI, suggesting a possible beneficial effect on body composition due to a correct development of the gut–adipose tissue axis. These findings reinforce the notion that early introduction of fresh MD foods may contribute to healthier growth trajectories, with a favorable impact on body composition, gut microbiota, and possibly CID risk.

In our study, higher alpha diversity and enrichment of short-chain fatty acid (SCFA) producers were found in the MD group, in contrast with the control group. This result allows us to speculate that microbial metabolites such as SCFA (butyrate, acetate, and propionate, derived from dietary fiber fermentation in the gut) may serve as indirect evidence of an eubiotic gut flora.

We are aware that, while encouraging, these preliminary results require confirmation in larger cohorts and through interventions extended beyond the first 3 years of life, including maternal adherence to the MD during pregnancy and breastfeeding. Extending dietary support during school years may also be critical. However, all together, our results support the hypothesis that early nutrition and complementary feeding may have an important role in shaping the gut microbiota and that early eubiosis stays up to adulthood, possibly leading to a lower incidence of CID.

Finally, it is worth emphasizing the public health implications of this project. Our study, as well as our previous work [7], was made possible by the frame of the Italian primary care pediatric system and the integration of the pediatric residency program in the daily clinical practice of family pediatricians [14]. This model demonstrates that large-scale, real-life prospective studies can be conducted within routine clinical practice, leveraging existing community pediatricians and routinely collected clinical data, thus reducing costs. If confirmed, this approach could represent a reproducible model for future randomized clinical trials [15] and for the development of feasible, low-cost preventive strategies for CID, as required by some authors [16,17].

We are aware that our study has some limitations. First, the sample size was relatively small, particularly for the microbiota analyses, which were restricted to a subset of 36 children, limiting statistical power and generalizability. Second, the dietary intervention was confined to the first years of life, with no structured nutritional education provided after 3 years, which may explain the convergence of overall MD adherence between groups at 9 years. In addition, the physiological dropout of patients reduced the number of children available for long-term follow-up, potentially introducing selection bias despite comparable baseline characteristics. The study was conducted in a single national context within the Italian pediatric primary care system, which may limit external validity to other healthcare settings or cultural environments. Despite these limitations, we still believe that our work may represent a valid model of research.

## 5. Conclusions

The results of this 9-year follow-up suggest that introducing fresh Mediterranean diet foods, prepared in a palatable way during complementary feeding, may exert lasting effects on gut microbiota composition and on certain dietary habits, even though overall MD adherence later converges between groups. The persistence in the MD group of greater microbial diversity and enrichment in SCFA-producing taxa suggests that early-life nutritional exposure may promote the development of a more eubiotic gut ecosystem, potentially associated with a lower risk of chronic diseases such as atopic dermatitis, allergic rhinitis, autism spectrum disorder, and ADHD. Although differences in CID incidence did not reach statistical significance, the favorable trends observed in the intervention group support the hypothesis of possible long-term benefits. These preliminary findings reinforce the concept that complementary feeding represents a critical window for preventive interventions and highlight the need for larger studies with extended follow-up. Finally, this study underscores the feasibility of conducting large-scale, real-life pediatric research through collaboration between primary care pediatricians and residency programs.

## Figures and Tables

**Table 1:** Demographic characteristics of the children at the time of evaluation.

	Overall (*n* = 191; 100%)	Control Group (*n* = 95; 49.7%)	MD Group (*n* = 96; 50.3%)
Gender			
Male	89 (46.6)	48 (50.5)	41 (42.7)
Female	102 (53.4)	47 (49.5)	55 (57.3)
Age at evaluation	7.8 ± 0.5 (4.8 to 8.8)	7.8 ± 0.4 (6.6 to 8.8)	7.8 ± 0.6 (4.8 to 8.6)

**Table 2:** Frequency of adherence to individual KidMed components.

	Overall (*n* =191; 100%)	Control Group (*n* = 95; 49.7%)	MD Group (*n* = 96; 50.3%)	*p*
Fruit or freshly squeezed fruit juice every day	152 (79.6)	80 (84.2)	72 (75)	0.151
More fruit per day	82 (42.9)	43 (45.3)	39 (40.6)	0.560
Fresh and cooked vegetables once a day	118 (61.8)	49 (51.6)	69 (71.9)	0.005
More fresh or cooked vegetables per day	57 (29.8)	24 (25.3)	33 (34.4)	0.206
Fish regularly (at least 2–3 times per week)	105 (55.3)	49 (51.6)	56 (58.9)	0.381
Fast-food more than once a week.	23 (12)	12 (12.6)	11 (11.5)	0.828
Legumes more than once a week	181 (94.8)	91 (95.8)	90 (93.8)	0.747
Pasta and rice almost every day	189 (99)	93 (97.9)	96 (100)	0.246
Breakfast bread or grain products (cereals)	85 (44.5)	39 (41.1)	46 (47.9)	0.383
Nuts regularly (at least 2–3 times per week)	43 (22.6)	21 (22.3)	22 (22.9)	>0.99
Olive oil at home	190 (99.5)	94 (98.9)	96 (100)	0.497
Eats Breakfast	164 (85.9)	79 (83.2)	85 (88.5)	0.307
Milk and dairy products for breakfast	133 (69.6)	67 (70.5)	66 (68.8)	0.875
Baked goods and pastries for breakfast	145 (75.9)	74 (77.9)	71 (74)	0.612
Yogurt and/or a large slice cheese	55 (28.8)	27 (28.4)	28 (29.2)	>0.99
Sweet sugar and sweets several times a day	64 (33.5)	30 (31.6)	34 (35.4)	0.646

**Table 3:** Anthropometric measurements.

	Overall (*n* = 191; 100%)	Control Group (*n* = 95; 49.7%)	MD Group (*n* = 96; 50.3%)	*p* Value
BMI; kg/m^2^	17.9 ± 3.4 (11.2 to 28.6)	17.7 ± 3.5 (11.8 to 28.5)	18.1 ± 3.4 (11.2 to 28.6)	0.384
Overweight; *n* (%)	47 (25)	21 (22.8)	26 (27)	0.613
Obese; *n* (%)	8 (4.3)	6 (6.5)	2 (2.1)	0.163

**Table 4:** Cumulative incidence of Inflammatory Chronic Diseases.

	Overall (*n* = 191; 100%)	Control Group (*n* = 95; 49.7%)	MD Group (*n* = 96; 50.3%)	*p* Value
Asthma	35 (21.2)	15 (18.1)	20 (24.4)	0.346
Constipation	7 (4.2)	5 (6)	2 (2.4)	0.443
Irritable Bowel Syndrome	1 (0.6)	0 (0)	1 (1.2)	0.497
Autism Spectrum Disorders	4 (2.4)	4 (4.8)	0 (0)	0.12
ADHD	6 (3.6)	4 (4.8)	2 (2.4)	0.682
Allergic Rhinitis	14 (8.5)	9 (10.8)	5 (6.1)	0.403
Eczema	1 (0.6)	1 (1.2)	0 (0)	>0.99
Atopic Dermatitis	15 (9.1)	9 (10.8)	6 (7.3)	0.59
Atopic Dermatitis or Allergic Rhinitis	27 (16.4)	16 (19.3)	11 (13.4)	0.401
Autism Spectrum Disorders or ADHD	10 (6.1)	8 (9.6)	2 (2.4)	0.099
At least one chronic disease	80 (48.5)	40 (48.2)	40 (48.8)	>0.99

## Data Availability

The raw data supporting the conclusions of this article will be made available by the authors on request.
